# In Vitro Fermentation of Sheep and Cow Milk Using Infant Fecal Bacteria

**DOI:** 10.3390/nu12061802

**Published:** 2020-06-17

**Authors:** Natalie Ahlborn, Wayne Young, Jane Mullaney, Linda M. Samuelsson

**Affiliations:** 1 AgResearch Ltd., Grasslands Research Centre, Palmerston North 4442, New Zealand; natalie.ahlborn@agresearch.co.nz (N.A.); Wayne.Young@agresearch.co.nz (W.Y.); jane.mullaney@agresearch.co.nz (J.M.); 2 Faculty of Life Sciences, Rhine-Waal University of Applied Sciences, 47533 Kleve, Germany; 3 Riddet Institute, Massey University, Palmerston North 4474, New Zealand; 4 High Value Nutrition, National Science Challenges, The Liggins Institute at the University of Auckland, Auckland 1010, New Zealand

**Keywords:** ovine milk, bovine milk, digestibility, in vitro digestion, infant gut microbiome, fecal fermentation, NMR, short-chain fatty acids, metabolomics

## Abstract

While human milk is the optimal food for infants, formulas that contain ruminant milk can have an important role where breastfeeding is not possible. In this regard, cow milk is most commonly used. However, recent years have brought interest in other ruminant milk. While many similarities exist between ruminant milk, there are likely enough compositional differences to promote different effects in the infant. This may include effects on different bacteria in the large bowel, leading to different metabolites in the gut. In this study sheep and cow milk were digested using an in vitro infant digestive model, followed by fecal fermentation using cultures inoculated with fecal material from two infants of one month and five months of age. The effects of the cow and sheep milk on the fecal microbiota, short-chain fatty acids (SCFA), and other metabolites were investigated. Significant differences in microbial, SCFA, and metabolite composition were observed between fermentation of sheep and cow milk using fecal inoculum from a one-month-old infant, but comparatively minimal differences using fecal inoculum from a five-month-old infant. These results show that sheep milk and cow milk can have differential effects on the gut microbiota, while demonstrating the individuality of the gut microbiome.

## 1. Introduction

Recent years have brought renewed interest in alternative milk such as sheep, goat, and camel milk as a substitute for cow milk in the diet, both for adults and infants [1,2,3]. Sheep milk is the fourth most consumed milk worldwide, making up 2.3% of global milk production [4]. One of the reasons for the increased interest in sheep and goat milk is the apparent tolerance of these milk by individuals with a cow milk intolerance, which has been linked to protein variation [4,5]. Despite the potential of sheep milk in the human diet, the effects of sheep milk on human health and the human gut microbiome have not been well reported.

The symbiotic relationship between the gut microbiota of each human and their human host means that the microbiota influences many areas of health and development beyond the gut. The presence of healthy, beneficial gut bacteria is of particular importance in the development of infants, as disruptions to the early gut microbiota are linked to immune problems, metabolic disorders, and adult health risk factors later in life [6]. Examples demonstrating microbiome involvement include the development of the enteric nervous system and an infant’s susceptibility to developing allergies, or tendencies towards neurodevelopmental disorders [6,7,8,9].

The composition of the gut microbiome can be directly influenced by diet [10]. A key shift in the infant microbiome occurs with the transition of human infants from mother’s milk to other mammal’s milk and eventually onto solid food. This diet transition is characterized by a shift in microbial composition towards species that are able to utilize the dominant contents of the new food type [11]. 

Sheep milk has several physicochemical and nutritional characteristics that distinguish it from cow milk, particularly in terms of protein, solids, lipids, and vitamins and minerals [4,5]. Feeding these milk for longer periods of time may affect both the microbiome and the body differently [12].

Previous digestive studies have examined the human digestion of cow and goat milk [2,5,13,14], but very few have compared the digestion and large bowel fermentation of cow milk with sheep milk, particularly in an infant in vitro model. This study was undertaken to analyze the difference between sheep milk and cow milk in terms of the digestion and large bowel fermentation in the human infant, using an in vitro model to simulate infant digestion, removal of nutrients in the small intestine and fermentation of remaining substrates by the large bowel microbiota.

To analyze the changes in microbial metabolism and composition as a result of the digestion of different kinds of milk, short-chain fatty acids (SCFA) concentrations were measured and 16S rRNA amplicon sequencing was carried out. As bacteria produce many other products such as organic acids and alcohols, which may be affected by the difference in the substrate, nuclear magnetic resonance (NMR) spectroscopy was used to detect and quantify other highly abundant metabolites produced during fermentation.

## 2. Materials and Methods

A three-stage process designed to simulate the gastrointestinal processing of sheep milk and cow milk by an infant was used to assess the differences induced by the two types of milk on the composition and metabolism of the fecal microbiome. The composition of the microbiome was assessed using 16S rRNA amplicon sequencing and SCFA and other metabolites were analyzed by GC-FID and NMR spectroscopy, respectively.

### 2.1. Materials

Sheep milk and cow milk used in this work were provided by commercial dairy farms and were frozen before use. Baby feces were donated and stored at −80 °C shortly after collection.

Chemicals used were as follows: KCl (99.5%), KH_2_PO_4_ (99.5%), MgCl_2_(H_2_O)_6_, orthophosphoric acid (85%), and diethyl ether (98%) were sourced from BDH Laboratory Supplies, Dorset, England. NaCl (99.6%) and CaCl_2_ (99%) were sourced from ThermoFisher Scientific, (Waltham, MA, USA). NaHCO_3_ (99%) was sourced from JT Baker, (Phillipsburg, NJ, USA). Pancreatin (37452 FIP-U/mg) was sourced from AppliChem GmbH, (Darmstadt, Germany). D_2_O (99.8%) was sourced from Cambridge Isotope Labs Inc, (Tewksbury, MA, USA). (NH_4_)_2_CO_3_ (99%), pepsin, gastric lipase (20,000 U/mg), cysteine (97%), bile salts, ethylbutyric acid (98%), N-methyl-N-t-butyldimethylsilyltrifluoroacetamide (95%), and imidazole (99%) were sourced from Sigma-Aldrich, (St. Louis, MO, USA).

### 2.2. In Vitro Digestion of Milk

Raw skimmed sheep and cow milk was subjected to in vitro digestion using a method based on Minekus, et al. [15] and combined with a dialysis step to remove small molecules released by the simulated digestion. Modifications include the exclusion of an oral digestion phase, as suggested by Minekus et al. (2014), due to the very short period of time a liquid meal spends in the oral cavity, and a pH of 3 to simulate infant digestive conditions. Raw cow and sheep milk were skimmed by centrifuging twice at 4000× *g* for 30 m with the fat skimmed from the top after each centrifugation. The pH of the skimmed milk was then adjusted to 3 at room temperature. Seven mL of simulated gastric fluid (SGF; 6.9 mM KCl, 0.9 mM KH_2_PO_4_, 25 mM NaHCO_3_, 47.2 mM NaCl, 0.1 mM MgCl_2_(H_2_O)_6_, 0.5 mM (NH_4_)_2_CO_3_, pH 3) was added to 10 mL of each milk, followed by 1 mL of pepsin stock solution (2000 U/mL in SGF). This was followed by the addition of 100 µL of 300 mM CaCl_2_ and 1 mL of gastric lipase solution (800 U/mL in SGF). Water was added to make a total volume of 20 mL. The milk digests were then incubated at 37 °C shaking for 2 h. To mimic the small intestinal phase of digestion, 11 mL of simulated intestinal fluid (SIF; 6.8 mM KCl, 0.8 mM KH_2_PO_4_, 85 mM NaHCO_3_, 38.4 mM NaCl, 0.33 mM MgCl_2_(H_2_O)_6_, pH 6.5), 2.5 mL of bile salt solution (16 mM bile salt in SIF, pH 7), 40 µL of 300 mM CaCl_2_, and 1.13 mL water was added to the resulting chyme. This was then incubated for 10 min at 37 °C in a shaker. Following incubation, 5 mL of pancreatin solution (4.33 g of pancreatin powder in 10 mL of SIF) was added and the resulting solution incubated at 37 °C for two h in a shaker. After incubation, the enzymes were heat-inactivated by microwaving on high for 1 min. The tubes were then cooled on ice and left in the fridge overnight. Following digestion, the milk types were dialyzed using 24 cm of dialysis tubing (molecular weight cut-off 100–500, diameter 31 × 20 mm, 3.1 mL per cm). The tubing was first soaked for 30 min in Milli-Q to remove any residual glycerol and glucose. Then, 40 mL of digest was added to the tubing, which was put into a 4 L vessel for dialysis. This was left for 24 h with water changes at 5, 9.5, and 21.5 h. The resulting retentate was aliquoted into Falcon tubes and frozen at −80 °C and was used as a substrate for batch fecal cultures to simulate the fermentation of these kinds of milk in the large bowel. 

### 2.3. Fecal Fermentation of Milk Digest Retentates

The cultures used as fermentation inoculates were extracted from fecal samples from two infants; a one-month-old infant, who had been fed exclusively on a breast milk diet, and a five-month-old infant, who had been breastfed and was transitioning onto solid food. This transition is reflected in changes to the microbiome as different substrates are introduced by diet variation [16]. To investigate the effects of feeding sheep and cow milk on infant fecal bacteria at different developmental stages, we used fecal samples from two unrelated infants of different ages. The method for fecal fermentation was based on the methods used by Edwards, et al. [17]. 

For the preparation of the milk digests for fermentation, a 200 mM phosphate buffer solution was prepared by adjusting 700 mL of 0.2 M Na_2_HPO_4_ solution to pH 7.22 using a 0.2 M KH_2_PO_4_ solution. This buffer was autoclaved. A 3% cysteine solution was prepared by dissolving 12 mg of cysteine in 400 mL of Milli-Q water. The solution was stored at 4 °C for 48 h. A control ‘digest’ was made up of water in the place of the milk digest. This was made up of 7 mL SGF, 11 mL SIF, and 22 mL Milli-Q water.

Nappies containing baby feces were collected and stored at −80 °C. Once the contents had thawed, the feces were added to a separation bag. 60 mL of phosphate buffer was added to give a rough 4:1 ratio (buffer: fecal sample, mL:g), and the bag was agitated to create a fecal slurry. 

Tubes for four-time points (0, 4, 7, and 24 h) for the sheep milk, cow milk, and the control were prepared in triplicate. Each tube was prepared with 1.25 mL of defrosted milk digest or control and 1.25 mL of 2× phosphate buffer. The tubes were autoclaved and degassed by bubbling N_2_ through the contents for 5 min. Before inoculation, 25 µL of the 3% cysteine solution was added to each tube as a reducing agent and left for 5–10 min and the inoculum then added. To inoculate the tubes, 1.5 mL of the fecal slurry was added to each tube to give an estimated fecal concentration of 10% (w/v). CO_2_ was added to the headspace of the tubes and the tubes were incubated for either 0, 4, 7, or 24 h at 37 °C.

**Figure 1:**
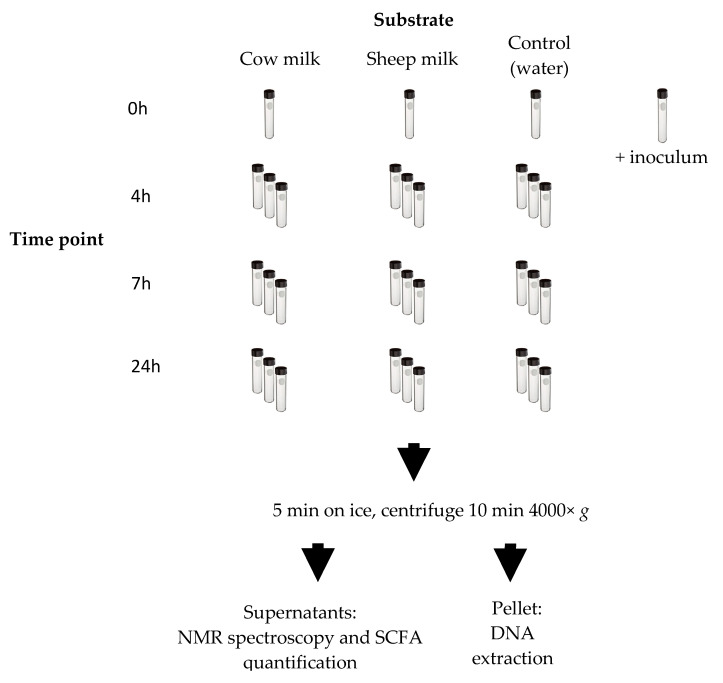
Fecal fermentation scheme. Each time point of the fermentation was done in triplicate except for the 0 h time point. A tube was prepared for the inoculum and was processed as the 0 h samples. Once each time point was reached, the appropriate number of tubes was removed from the warm room and fermentation stopped on ice. The ferments were centrifuged. The resulting supernatants were used for nuclear magnetic resonance (NMR) spectroscopy and short-chain fatty acids (SCFA) analysis by gas chromatography (GC). The resulting pellet was used for DNA extraction and sequencing.

Fermentation dynamics were assessed by sampling at different time points (0, 4, 7, and 24 h). These fermentation time points were selected as practical windows into the colonic digestion of milk, giving a good representation of changes to the microbiome and metabolites concentrations as substrates are progressively utilized, depending on their complexity. The utilization of dairy substrates over similar time frames have been shown in vivo [18,19]. Separate tubes were prepared for each time point to allow for triplicate sampling throughout the fermentation without disturbing the culture or changing the headspace conditions of the continued fermentation (Figure 1). The fermented liquid of the samples at each time point was cooled on ice for five min, then centrifuged at 4000× *g* for 10 min at 4 °C. The resulting supernatant was used for the quantification of short-chain fatty acids and analysis by NMR spectroscopy. The pellet was stored at −80 °C and used for DNA extraction.

### 2.4. Sample Analysis

Differences in microbial metabolism and composition of the gut microbiome resulting from the different digested kinds of milk were assessed by measuring SCFA concentrations using gas chromatography (GC), NMR-based metabolomics, and 16S rRNA gene amplicon sequencing. 

#### 2.4.1. SCFA Analysis

Analysis of SCFA by GC was carried out following a previously published method by Richardson, et al. [20]. The analysis was carried out using a Shimadzu GC-2010 Plus gas chromatograph (Shimadzu, Kyoto, Japan) with a barrier ionization detector (helium ionization) and a 30 m × 0.53 mm I.D. × 50 μm film MXT-Msieve5A PLOT capillary column (Restek Corporation, Bellefonte, PA, USA). A split injection of a 1 µL sample was made at a ratio of 5:1, with a column helium flow rate of 1.36 mL/min (total flow: 11.2 mL/min). Injector and detector temperatures were both 240 °C. The column temperature was initially held at 50 °C for 2 min and then increased by 5 °C/min to 130 °C, followed by 15 °C/min to 240 °C (held for 4.7 min). Run time per sample was 30 min. Following this, the resulting data were analyzed using a two-way permutation ANOVA in R [21], with time and treatment as factors. Permutation ANOVA is a non-parametric test that does not require data to be normally distributed.

#### 2.4.2. DNA Sequencing

DNA was extracted from culture pellets using NucleoSpin^®^ Soil kits (Machery-Nagel, Dueren, Germany) with the manufacturer’s protocol modified through the addition of a bead-beating step using a BioSpec Products Mini-Beadbeater 96 (Biospec Products, Bartesville, OK, USA) for 4 min @ 2100 oscillations/min, and an extra wash step. The extracted metagenomic DNA was sequenced by the Massey Genome Service (Massey University, Palmerston North, New Zealand) using Illumina MiSeq paired-end 2 × 250 bp amplicon sequencing of the V3 to V4 region of the bacterial 16S rRNA gene [22]. Sequence reads were processed using QIIME 1.8 [23]. Paired-end reads were quality filtered using default settings and sequences were chimera-checked using the USEARCH method against the Silva database (release 128). Sequences identified as chimeric were removed. Sequences were clustered at 97% similarity into operational taxonomic units (OTUs) using the UCLUST method. Representative sequences were assigned taxonomies using the Silva 128 database, and OTU’s were then grouped according to taxonomic level (phylum, family, order, class, and genus) for further analysis. Following this, the resulting data were analyzed using principal coordinate analysis (PCoA) and a two-way permutation ANOVA in R [21], with time and treatment as factors. 

#### 2.4.3. NMR Metabolomics

For NMR spectroscopy, supernatants from the fecal fermentation were defrosted on ice over 2 h, with intermittent vortexing. Samples were then filtered through a 0.2 µm syringe filter and 630 µL of supernatant was mixed with 70 µL internal standard solution (containing 100 mM imidazole and 5 mM DSS (2,2-dimethyl-2-silapentane-5-sulfonate) in deuterated water (D_2_O,)) in 5 mm NMR tubes. A pooled quality control (QC) sample was created by combining 25 µL of each supernatant. 1-dimensional (1D) ^1^H-NMR spectra were acquired for each fermentation supernatant sample on a Bruker 700 MHz Ultrashield NMR spectrometer (Bruker BioSpin GmbH, Rheinstetten, Germany) at Massey University, Palmerston North, using water suppression to suppress the large water peak. The noesypr1d pulse sequence was used with a recycle delay of 1.5 collecting 256 scans. A 90° pulse of 17.82 µs (at 7.2 dB) was used. 1D ^1^H-NMR and 2-dimensional (2D) NMR spectra (^1^H,^1^H- Total Correlation Spectroscopy (TOCSY), ^1^H,^13^C- Heteronuclear Single Quantum Correlation (HSQC)) were recorded on the QC sample for metabolite identification. Fermentation supernatant 1D ^1^H-NMR spectra were processed, and metabolites putatively identified and quantified using the Chenomx NMR Suite Professional 7.7 (Chenomx Inc., Edmonton, AB, Canada) software: phasing and baseline correction were performed and the pH was calibrated using the resonances from imidazole. The spectra were referenced to the DSS methyl peak at 0.00 ppm which was also used as an internal standard for quantitation. MetaboMiner [24,25], MestreNova v. 12.0.3 (MestreLab Research, Bajo, Spain) and COLMARm [26,27] were used to confirm the identity of the metabolites from the HSQC and TOCSY spectra. Peaks found were referenced against The Human Metabolome Database (HMDB) [28,29] or the Biological Magnetic Resonance Databank (BMRB) [30,31]. The metabolite data were pre-processed using a log transformation and Pareto scaling. A principal component analysis (PCA) was carried out using MetaboAnalyst [32,33], followed by a statistical analysis using a two-way ANOVA analysis in R, with time and treatment as factors. 

## 3. Results and Discussion

### 3.1. 16s rRNA Amplicon Sequencing

**Figure 2:**
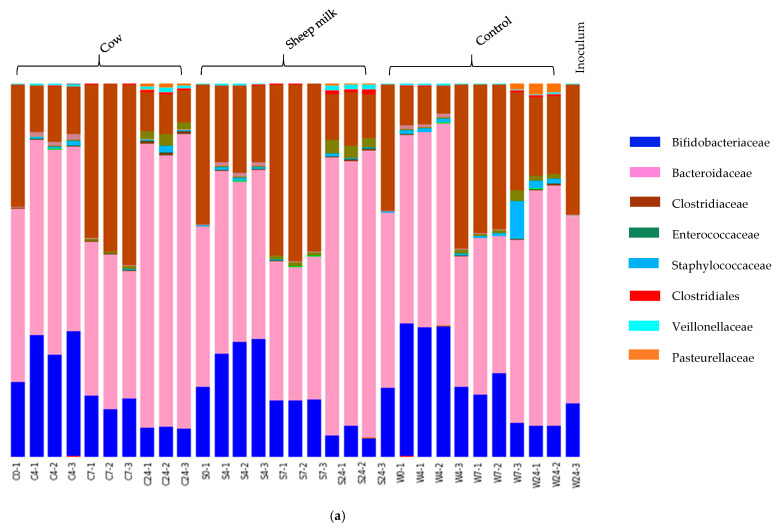

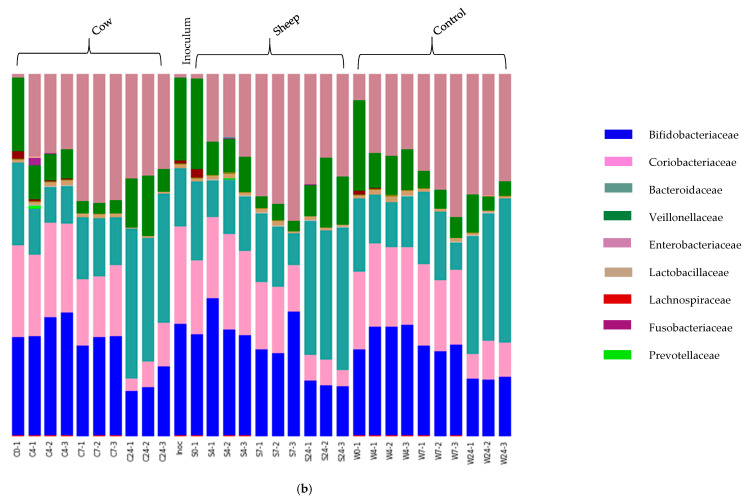
(**a**) One-month old infant faecal fermentation – taxonomic compositions of microbial populations at family level. Bars are labelled by treatment (sheep milk, cow milk or water (control), followed by time point (0, 4, 7 and 24 h). Two of the dominant families showed significant differences in abundance between sheep milk and cow milk fermentations. These were bacteria of family Bacteroidaceae (genus Bacteroides) and family Clostridiaceae (genus Clostridium sensu stricto 1). (**b**): Five-month old infant faecal fermentation – taxonomic compositions of microbial populations at family level. The bars are labelled by treatment, followed by time point (0, 4, 7 and 24 h). The dominant families present throughout the fermentation are shown, along with some minor families. The initial dominant bacterial families inhibiting the gut remain dominant throughout the fermentations, although relative abundances of each vary with time and substrate.

Fecal fermentations from both the one-month-old and the five-month-old baby showed a clear development of distinct microbial communities over the four-time points (Figure 2a,b). The one-month-old baby fecal microbiota was characterized by Bacteroidaceae (45.32%), Clostridiaceae (34.78%) and Bifidobacteriaceae (19.07%) at time 0 (Figure 2a), whereas the five-month-old baby fecal microbiota consisted mainly of Bifidobacteriaceae (27.5%), Coriobacteriaceae (23.4%), Bacteroidaceae (20.3%), and Veillonellaceae (23.3%) at time 0 (Figure 2b). Over time, the relative abundance of Bifidobacteriaceae decreased in cultures from both infants; however, the taxa that increased correspondingly varied between the two infants.

In the one-month-old infant, the decrease of Bifidobacteriaceae was accompanied by an increase in Clostridiaceae and Bacteroidaceae, with the precise degree of change dependent on incubation time. On the other hand, in fermentations inoculated with the five-month-old feces the loss of Bifidobacteriaceae over time corresponded with an increase in Enterobacteriaceae and Bacteroidaceae.

**Figure 3:**
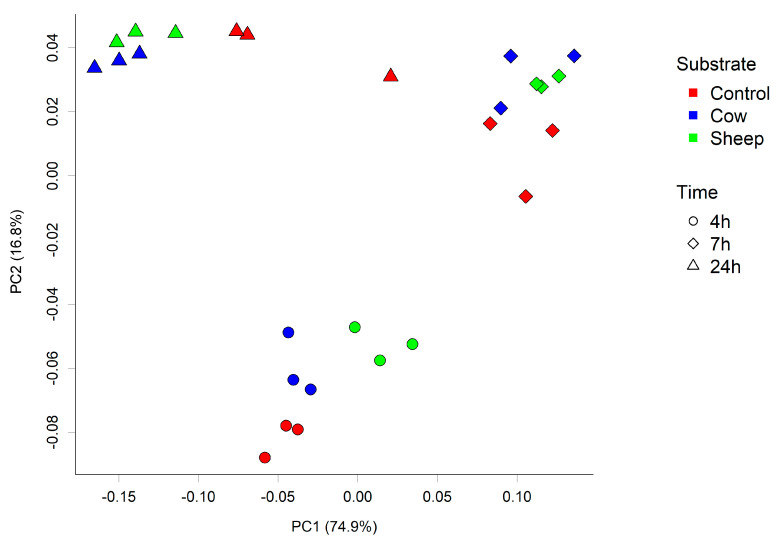
Principal coordinate analysis (PCoA) plot of the fecal microbial community from one-month-old child cultured with cow or sheep milk over 4, 7, and 24 h. PC1 and PC2 refer to principal coordinates 1 and 2 respectively.

In addition, differences in microbial composition were observed between cultures with sheep or cow milk added as a substrate in fermentations seeded with fecal material from the one-month-old baby. A PCoA plot of the community weighted Unifrac phylogenetic distances showed the greatest variation in cultures was along PC1, which is likely due to the difference between time points (Figure 3). Cultures here are grouped separately by time point, however, a secondary grouping of cow milk and sheep milk can be seen at 4 h, but is less defined at 7 h and 24 h (Figure 3). At 4 h, differences included significantly greater abundance of Clostridiaceae (genus *Clostridium sensu stricto 1*) in sheep milk cultures than in cow milk cultures (*p* = 0.049; 21.6% and 13.3% respectively) and significantly lower abundance of Bacteroidaceae (genus *Bacteroides*) (*P* < 0.049; 45.9% and 52.2% respectively) (Figure 2a). At 7 h and 24 h the abundance of *Clostridium Sensu stricto* 1 was consistently higher in sheep milk cultures although the difference was less pronounced (7 h; 46.1% and 44.9% respectively, 24 h; 12.7% and 9.8% respectively). The abundance of *Bacteroides* was consistently lower in sheep milk cultures than cow milk cultures at 7 h and 24 h (7 h; 37.0% and 38.9% respectively, 24 h; 74.0% and 75.9%). 

In contrast, fecal cultures from the five-month-old infant showed very little differences in microbiota composition between those with cow or sheep milk; however, the addition of either milk type did result in differences compared to cultures with no added milk. This included a significantly higher relative abundance of Veillonellaceae in cultures with milk after 24 h.

The infant gut microbiota has previously been categorized by the composition and occurrence of main bacterial groups into six main phylogenetic groups by Vallès, et al. [34] and further discussed by Milani, et al. [35]. The dominant bacteria in different individuals are reportedly represented by *Bifidobacterium*, *Veillonella*, *Streptococcus*, *Citrobacter*, *Escherichia*, *Bacteroides,* and *Clostridium*, with minor genera including *Bacillus*, *Enterococcus*, *Eubacterium*, *Lactobacillus*, *Prevotella,* and *Ruminococcus*. Thus the core bacteria of the infant gut microbiota include members of group 1 (*Enterobacteriales: Escherichia*), group 2 (*Bacteroidales: Bacteroides* and *Prevotella*), group 5 (*Clostridiales: Clostridium* and *Eubacterium*), and group 6 (*Bifidobacteriales*: *Bifidobactrium; Lactobacillales: Enterococcus*, *Lactobacillus,* and *Streptococcus; Clostridiales: Ruminococcus*) [34]. Both the one-month-old infant and the five-month-old infant starter microbiomes are consistent with the reported groups in terms of dominant bacteria. 

A study in mice showed a diet high in *n*-3 polyunsaturated fatty acids (PUFA) increases the abundance of *Clostridium sensu stricto* [36,37]; this supports our findings of higher abundances of these bacteria in the sheep milk fermentations as *n*-3 PUFA are found in higher concentrations in sheep milk than in cow milk [38]. *Clostridium sensu stricto* are a major component of the infant fecal microbiome [39,40], and disruption of the relative abundance of these bacteria, amongst other groups, can have implications on health. In children, lower abundances of *Bacteroides*, *Parabacteroides,* and *Veillonella* and corresponding higher abundances of *Clostridium sensu stricto* (among other bacterial groups) have been associated with children with food sensitization issues, when compared to healthy controls [41]. In conjunction with this, a positive correlation between high abundances of *Clostridium sensu stricto* and serum specific Ig-E antibodies has been observed, implicating the involvement of these bacteria in the development of IgE-mediated food allergies [42]. 

*Bacteroides* are commensal anaerobic microbes found in the human gut, where the delivery mode is a key driver of abundances of *Bacteroides* in the infant and reduced levels are seen in infants delivered via cesarean section [9,40]. Following delivery, abundances appear to be largely modulated by human milk oligosaccharides, however, *Bacteroides* can easily adapt to the nutritional conditions of their host and utilize both host-derived glycans as well as dietary polysaccharides, and can also incorporate external amino acids [43]. They are thought to be the largest propionate producers in the human gut [44] and correlations between the abundance of *Bacteriodes* and fecal SCFA levels have been observed in mice, using antibiotics to modulate the microbiome composition [45].

The greater abundance of *Clostridium sensu stricto* in the sheep milk fermentations compared to cow milk fermentations suggests that particular components in sheep milk promote the growth of these bacteria. While the abundance of *Bacteroides* was lower in the one-month-old sheep milk fermentations the results from the five-month-old baby showed no difference in the abundance of *Bacteroides* between sheep milk and cow milk fermentations. This indicates that milk type alone is not responsible for altering the activity of *Bacteroides*; rather the decrease in Bacteroides could be associated with a corresponding increase in *Clostridium sensu stricto* in the sheep milk fermentations.

In a fecal fermentation study using a variety of cereal additions to infant diets, Gamage (2007) showed an increase in the abundance of Veillonellaceae with the addition of oats, which is in line with other studies reporting similar results after the introduction of foods high in complex carbohydrates [46,47]. It is likely that the higher abundances of these bacteria, such as those found in the cow milk fermentations, will aid the successful transition of an infant from a milk-based diet to a solid-food diet.

### 3.2. NMR-Based Metabolomics

**Figure 4:**
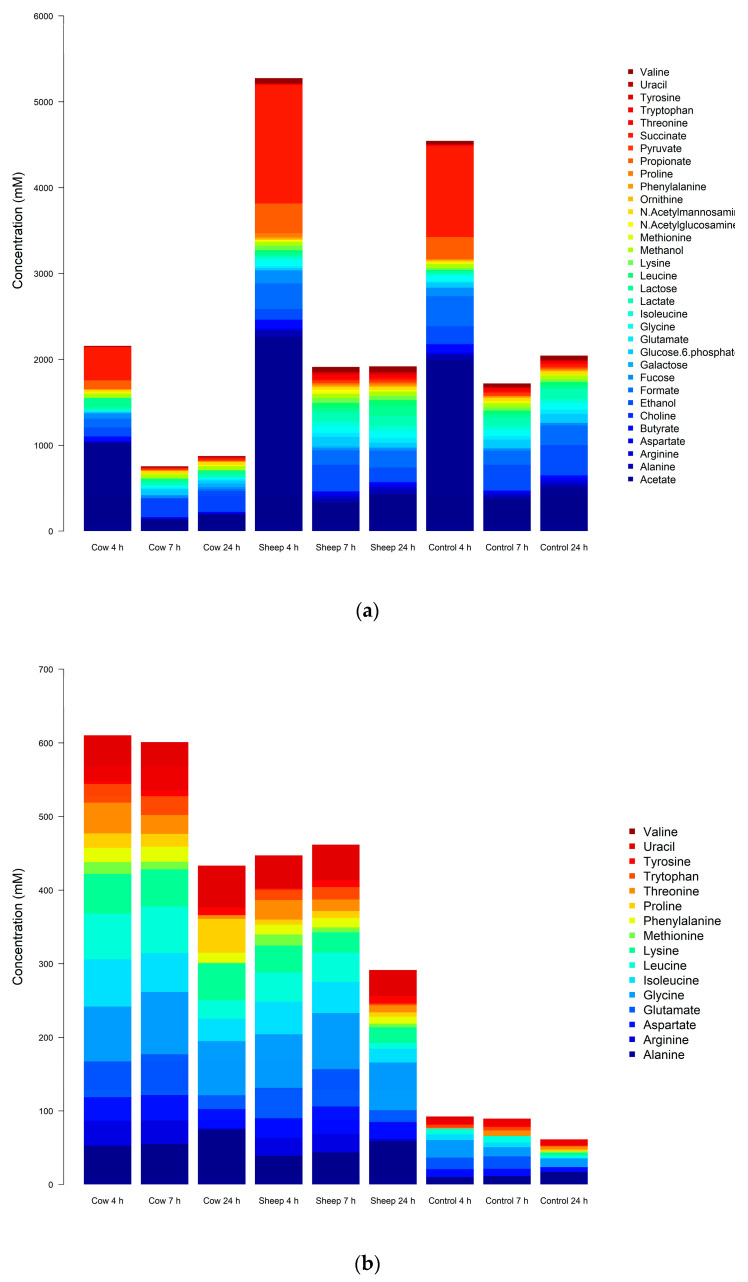

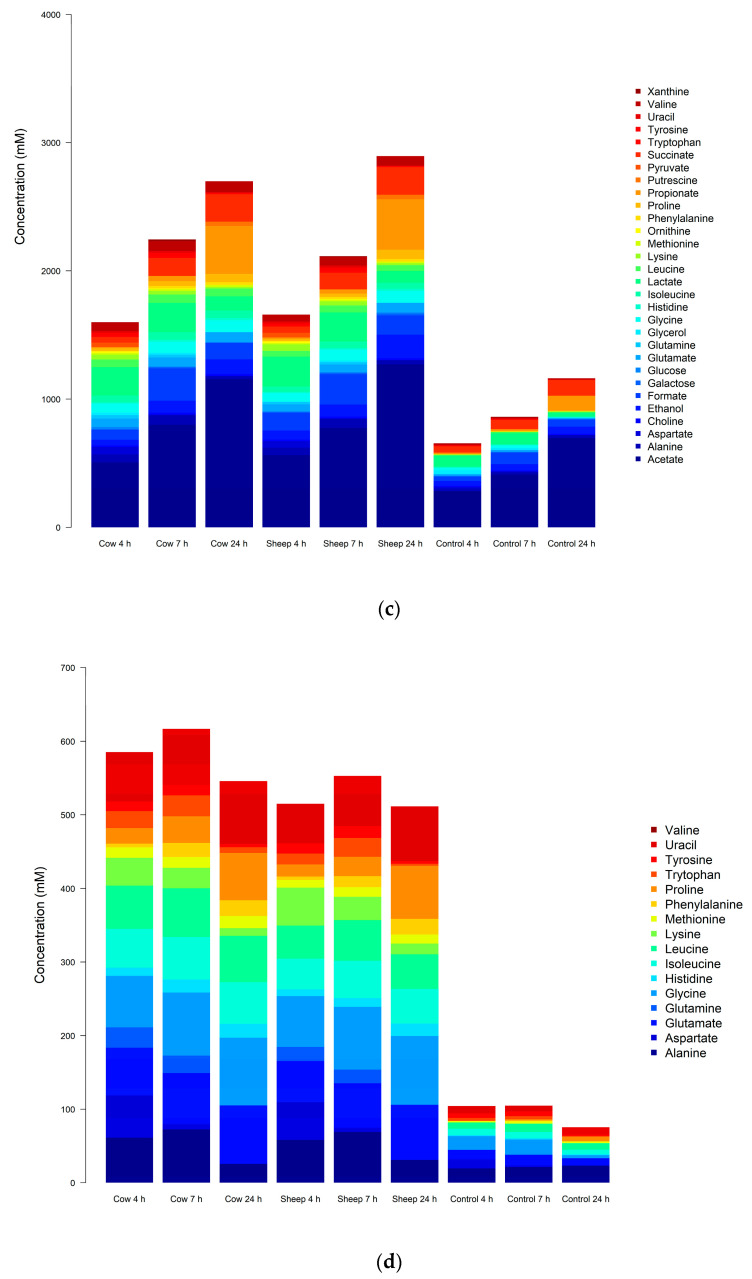
(**a**): One-month-old infant fecal fermentations—all metabolites with significantly different concentrations between all treatment groups, detected by NMR spectroscopy. (**b**): One-month-old infant fecal fermentations—amino acids with significantly different concentrations between all treatment groups, detected by NMR spectroscopy. (**c**): Five-month-old fecal fermentations—all metabolites with significantly different concentrations between all treatment groups, detected by NMR spectroscopy. (**d**): Five-month-old fecal fermentations—amino acids with significantly different concentrations between all treatment groups, detected by NMR spectroscopy.

In the one-month-old fermentations, 34 metabolites were identified and quantified in the NMR spectra (Table A1 and Table A3). An ANOVA showed 21 metabolites with significantly different concentrations between fermentation of sheep milk and cow milk (Table A4, Figure 4a–b). This included 10 amino acids (alanine, phenylalanine, tryptophan, proline, lysine, tyrosine, arginine, isoleucine, leucine, and valine), 6 sugars (*N*-acetylmannosamine, lactose, galactose, fucose, *N*-acetylglucosamine, and glucose-6-phosphate), 3 SCFAs (formate, propionate, and succinate), choline and ethanol (Figure 4a). 

In comparison, the 41 metabolites confirmed present in the five-month-old fermentations included 21 amino acids, 4 sugars, 5 short-chain fatty acids, 4 alcohols, 1 vitamin, 1 ketone, 1 polyamine, and 3 pyrimidine bases (including one derivative) (Table A2 and Table A5). Of these, eight metabolites were significantly different (*p* < 0.05) between cultures with digested sheep milk and digested cow milk (Table A6, Figure 4c–d).

These were the three amino acids methionine, glutamine, and histidine; the three branched-chain amino acids leucine, valine, and isoleucine; acetate (a short-chain fatty acid) and xanthine (a purine base).

**Figure 5:**
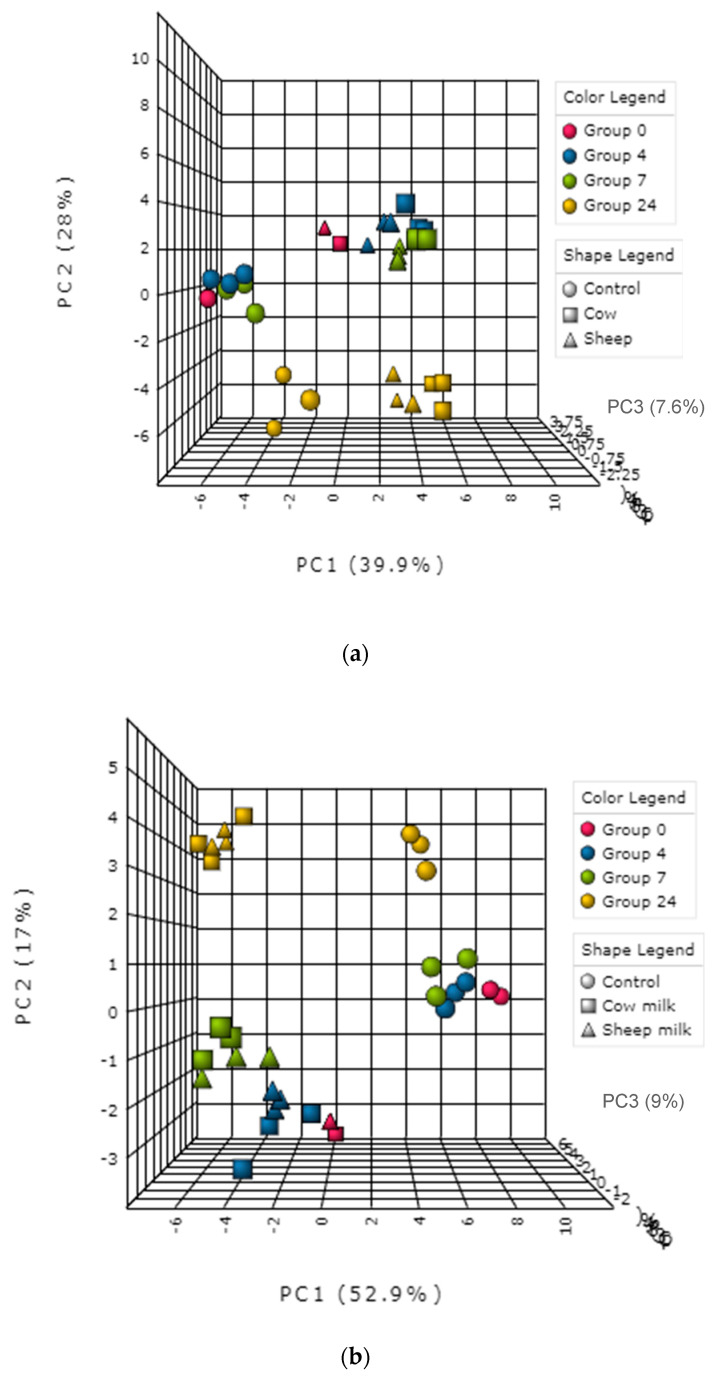
(**a**): Principal component analysis (PCA) plot—metabolite profile of the one-month old’s fecal fermentation, analyzed using NMR metabolomics. (**b**): PCA plot—metabolite profile of the five-month old’s fecal fermentation, analyzed using NMR metabolomics.

Initially, a PCA scores plot showed separations of the one-month old’s fecal fermentation metabolite profiles by time and between the control and milk fermentation, however, further distinct groupings of cow milk and sheep milk can be seen within the overarching 4 h fermentation group (Figure 5a). A PCA scores plot of the five-month old’s fermentations shows similar results (Figure 5b). 

The amino acid compositions of sheep and cow milk have been well characterized, such as in the following work by Claeys, et al. [48] and Kuiken and Pearson [49]. While the amount of each amino acid varies between sheep and cow milk, the proportions of each amino acid to total milk protein are very similar [48].

In both the one-month and the five-month old’s fecal fermentations, the concentrations of all the significantly different amino acids (Table A4 and Table A6) were lower in the 4 h and 7 h sheep milk fermentation than in the cow milk fermentations (Figure 4b,d). This could indicate that the protein in sheep milk, once digested and fermented in the gut, releases less amino- and branched-chain amino acids. However, an alternative explanation may be that proteins in sheep milk are instead digested and absorbed earlier in the digestion process than cow milk proteins. During digestion, the proteins in milk are enzymatically broken down into free amino acids or di- or tri-peptides, which can then be absorbed directly into the bloodstream from the small intestine. Thus, degradation of a larger proportion of proteins in the stomach likely results in more amino acids being absorbed in the small intestine (here mimicked by dialysis) and less of these entering the large intestine for fermentation. A study by Montoya, et al. [50] on gastric beef protein digestion and small intestine amino acid absorption in piglets found that proteins already largely digested in the stomach will be absorbed in the first half of the small intestine. This supports our observations and may indicate that sheep milk could be easier to digest than cow milk, allowing more amino acids to be absorbed early in the digestion process, offering faster utilization by the body. This is especially relevant for the essential amino acids and BCAAs (leucine, isoleucine, lysine, histidine, methionine, phenylalanine, tryptophan, threonine, and valine), where a fast absorption from muscle tissue is beneficial for quick release energy metabolism or individuals with high protein requirements, for example in athletes or the ill [51]. Except for tyrosine and arginine at 24 h in the one-month-old fermentations, the other amino acid concentrations at 24 h are also all lower in the sheep milk fermentation than in the cow milk fermentations. The bacterial utilization of amino acids may also contribute to the decrease in concentrations of most amino acids, as seen clearly at 24 h (Figure 4b,d).

### 3.3. Short-Chain Fatty Acids by Gas Chromatography

Short-chain fatty acids produced during colonic digestion have several roles within the body, including in energy metabolism, gut health, and immune regulation [52,53,54,55,56]. 

**Figure 6:**
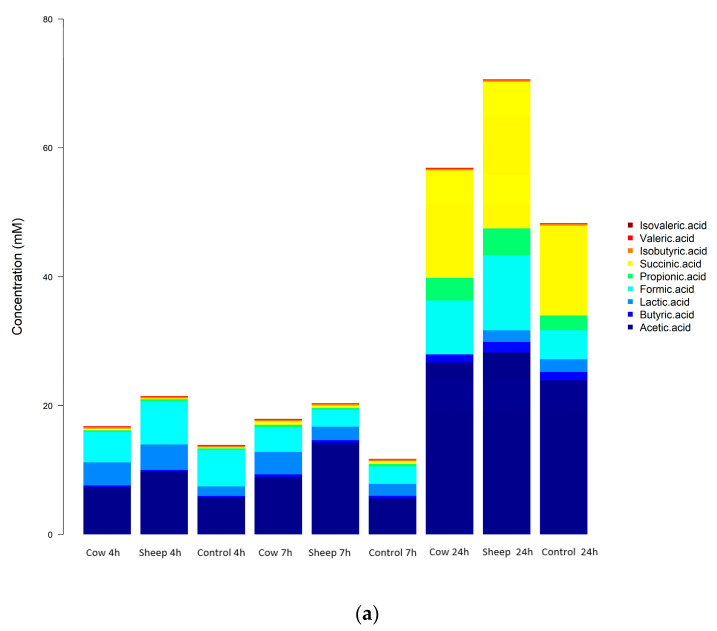

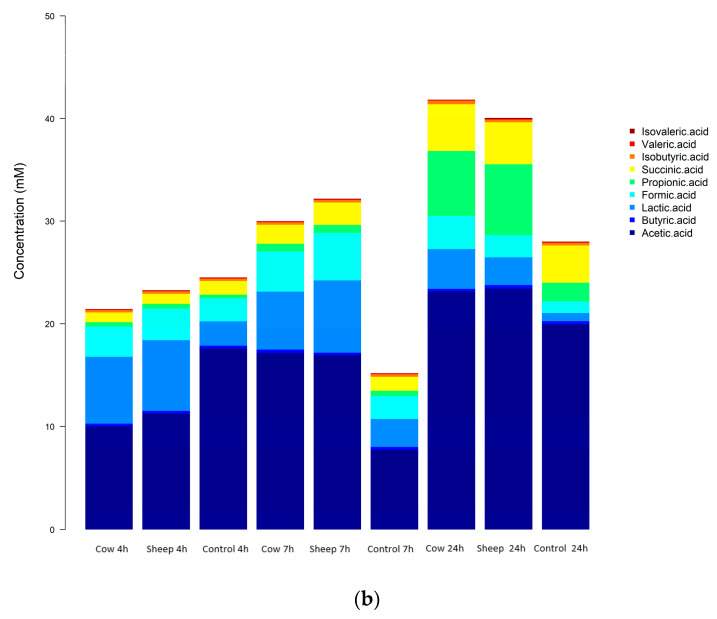
(**a**): One-month-old infant fecal fermentation—short chain fatty acids detected by gas chromatography. (**b**): Five-month-old infant fecal fermentation—short-chain fatty acids detected by gas chromatography.

Eight out of eleven measured SCFAs were detected in both fecal fermentations using GC. These were acetic acid, butyric acid, formic acid, isobutyric acid, isovaleric acid, lactic acid, propionic acid, and succinic acid (Figure 6a,b). Hexanoic, heptanoic and valeric acid were not detected in any fermentation. 

In the one-month old’s fecal fermentations, concentrations of acetic acid were significantly higher (*p* < 0.05) in sheep milk fermentations than cow milk fermentations, across all time points (Figure 6a). Both sheep and cow milk fermentations also had significantly higher concentrations of acetic acid than the control fermentations across all time points.

In the five-month-old fermentations, only isobutyric acid showed a significant difference (*p* < 0.05) in concentration when comparing sheep milk fermentations to cow milk fermentations. Isobutyric acid concentrations were the same at 4 h and 7 h in both sheep milk and cow milk fermentations, however at 24 h the concentration increased in the cow milk fermentation while remaining the same in the sheep milk fermentations (Figure 6b).

In both infant fecal fermentations, an increase in concentrations of acetic, propionic, and succinic acids and decrease of lactic acid concentrations with time was observed, indicating a classic mixed-acid model of fermentation. The production of these SCFAs is representative of a common gut SCFA profile and is supported by the presence of some key fermenters identified by the 16S sequencing. In the one-month-old, the large increase in the concentrations of acetate, succinate, and propionate particularly at 24 h, with higher concentrations in cow milk fermentations than sheep milk fermentations aligns well with the significant increase observed in the abundance of *Bacteroides* (cow milk; 38.9% at 7 h to 75.9% at 24 h). *Bacteroides* typically produce these SCFAs during fermentation, however other bacteria also produce these and other SCFAs either through the direct utilization of undigested dietary polysaccharides or amino acids and proteins or by metabolic cross-feeding [43]; hence, other types of fermentation cannot be ruled out. For example, the changes in concentrations of acetate throughout the sheep milk fermentations also corresponded with the relative abundance of Veillonellaceae. Members of the *Veillonella* genus have been shown to produce acetate and propionate via lactate fermentation [57,58]. 

The bacterial utilization of amino acids may also contribute to the decrease in concentrations of most amino acids, as seen clearly at 24 h (Figure 6a,b).

These data, together with the NMR metabolomics data and the comparison with the control fermentations show that there are components of the kinds of milk that continue to the large bowel where they are fermented, and not all components are removed in the ‘small intestine’ dialysis step. 

## 4. Conclusions

The fermentation of sheep and cow milk using the fecal microbiota of a one-month-old and a five-month-old infant showed several significant differences in microbial and metabolite composition between controls and different milk types. This indicates that components of the two kinds of milk escape into the large intestine with enough variation between kinds of milk to differentially modify the infant fecal microbiome. In conjunction with this, these results from two unrelated infants at different developmental stages show the role of an individual’s starter microbiome on the impact of feeding different kinds of milk, and effect on any potential health benefits that these kinds of milk could offer. 

Both milk types were observed to promote the development of bacterial families that digest complex carbohydrates and increase the production of SCFAs; therefore, the consumption of either type of milk by the infant as a host may be beneficial in preparing the gut for a transition to solid foods.

Metabolite profiles showed significant differences in the concentrations of some key amino acids between fermentation of sheep and cow milk. These data may indicate that sheep milk proteins are easier to digest than cow milk proteins, releasing amino acids early in the digestion process to allow absorption directly from the small intestine. 

A host’s gut microbiome is very specific to that individual, therefore the results and conclusions from this study are in the context of the two infants involved. To improve the understanding of the influence of sheep milk and cow milk on the general gastrointestinal microbiota, a follow-on study using a larger sample group (more infants) would be of value.

## Appendix A

Observed ^1^H and ^13^C NMR chemical shifts for quantified metabolites (referenced to DSS at 0.00 ppm) in human infant fecal fermentations following in vitro milk digestion. Metabolites that could not be confidently identified using the HSQC spectrum alone were confirmed using the TOCSY spectrum. Putatively identified metabolites whose presence was confirmed with the HSQC spectrum but could not be confirmed with the TOCSY spectrum are marked with an asterisk (*). Metabolite identities are considered correct if the proton and carbon shifts are within 0.03 and 0.3 ppm, respectively compared to the Human Metabolome Database.

**Table A1:** One-month-old infant fecal fermentations.

Metabolite	^1^H Chemical Shift	^13^C Chemical Shift	Confirmed TOCSY Cross-Peaks
**Acetate**	1.91	26.0	-
**Alanine**	3.77	53.4	-
	1.47	19.0	
**Arginine**	3.75	57.5	1.64/1.91
	3.23	43.4	1.90/3.76
	1.90	30.4	1.64/3.23
	1.65	26.6	1.64/3.77
**Aspartate**	3.90	55.1	2.79/3.89
	2.79	39.3	2.67/3.88
	2.68	39.3	2.79/2.67
**Butyrate**	0.88	16.0	-
	1.55	22.1	
	2.15	42.3	
**Choline**	3.19	56.6	3.51/4.05
	3.51	70.3	
	4.07	58.6	
**Ethanol**	1.17	19.5	-
	3.65	60.3	
**Formate**	8.45	173.8	8.45/8.45
**Fucose***	1.23	18.2	4.55/3.43 1.24/3.78
	3.43	74.4	
	3.62	75.6	
	3.73	74.2	
	3.79	73.6	
	4.54	98.9	
**Galactose**	3.72	63.9	
	3.80	71.1	
	3.84	71.9	-
	3.97	72.0	
	4.08	73.2	
	5.25	95.0	
**Glutamate**	2.12	29.6	3.75/2.08
	2.34	36.2	3.75/2.35
	3.75	57.5	2.08/2.34
**Glucose-6-phophate ***	3.58	74.0	5.22/3.57 5.22/3.55 5.22/3.72 5.22/4.03
	3.58	71.8	
	3.75	74.8	
	3.85	73.8	
	3.89	65.7	
	5.22	94.6	
**Glycine**	3.55	44.4	-
**Isoleucine**	0.93	13.8	-
	1.0	17.4	
	1.25	27.2	
	1.46	27.1	
	1.97	38.7	
	3.67	62.5	
**Lactate**	1.32	22.8	
	4.10	71.2	
**Lactose**	3.54	73.9	5.22/3.82 5.22/3.86 5.22/3.92 5.22/3.94 4.44/3.82 4.44/3.92 3.72/3.57 3.76/3.54 3.54/3.76
	3.58	74.0	
	3.64	81.0	
	3.65	75.3	
	3.75	77.9	
	3.77	63.8	
	3.82	74.3	
	3.88	62.5	
	3.90	71.2	
	3.95	72.9	
	4.43	105.7	
	5.22	94.6	
**Leucine**	0.94	23.6	
	0.95	24.7	
	1.69	26.8	-
	1.72	42.6	
	3.73	56.3	
**Lysine**	1.43	24.0	
	1.49	24.0	
	1.71	29.2	-
	1.89	32.7	
	3.02	412.1	
	3.75	57.5	
**Methanol**	3.35	51.8	-
**Methionine**	2.11	16.7	2.12/3.85 2.11/2.63
	2.17	32.5	
	2.63	31.5	
	3.85	56.8	
**N-acetyl glucosamine**	2.04	25.0	5.19/3.76 5.19/3.82 5.19/3.85 3.75/3.48
	3.47	72.6	
	3.75	73.2	
	3.82	63.4	
	3.82	75.3	
	3.85	56.8	
	5.20	93.7	
**N-acetyl mannosamine**	2.03	25.0	3.50/4.42 3.84/4.42 2.06/3.42 2.06/3.86
	3.40	72.6	
	3.53	73.2	
	3.80	63.4	
	3.84	75.3	
	4.44	56.8	
	5.02	93.7	
**Phenylalanine**	3.12	39.2	
	3.27	39.2	
	3.95	58.7	-
	7.32	132.1	
	7.37	130.5	
	7.42	131.9	
**Proline**	1.99	26.5	
	2.06	31.7	
	2.34	31.7	-
	3.33	49.0	
	3.41	49.0	
	4.12	64.0	
**Propionate**	2.17	33.4	-
	1.04	12.9	
**Pyruvate**	2.36	29.2	-
**Succinate**	2.40	36.8	-
**Threonine**	1.32	22.2	
	3.6	63.3	-
	4.25	68.8	
**Tryptophan**	4.05	58.0	7.73/7.53 7.27/7.53 3.29/4.04 3.47/3.30
	7.19	122.3	
	7.27	124.8	
	7.32	128.0	
	7.53	114.8	
	7.73	121.2	
**Tyrosine**	3.03	38.3	3.17/3.02 7.17/6.88
	3.18	38.3	
	3.95	58.7	
	6.89	118.7	
	7.18	133.6	
**Uracil**	7.52	146.3	7.52/5.79
	7.49	146.1	
	5.79	103.8	
**Valine**	0.98	19.4	
	1.03	20.7	-
	2.26	31.9	
	3.60	63.3	
***myo*-Inositol**	3.27	77.2	3.27/3.53 3.27/4.05 3.53/4.05 3.61/3.27 3.61/4.05
	3.53	73.9	
	4.05	75.0	

**Table A2:** Five-month-old fecal fermentation.

Metabolite	^1^H Chemical Shift	^13^C Chemical Shift	Confirmed TOCSY Cross-Peaks
**5,6-dihydrouracil**	2.67	32.27	2.67/3.44
	3.44	38.32	
**Acetate**	1.91	26.0	-
**Alanine**	3.77	53.4	-
	1.47	19.0	
**Arginine**	3.75	57.5	1.71/1.89
	3.22	43.6	1.86/3.75
	1.87	30.6	1.71/3.23
	1.68	26.5	1.71/3.73
**Aspartate**	3.89	55.1	3.90/2.81
	2.81	39.3	3.90/2.67
	2.67	39.4	2.80/3.89
			2.81/2.66
			2.66/3.89
			2.67/2.80
**Betaine**	3.88	69.8	3.88/3.23
	3.25	56.1	
**Choline**	3.19	56.6	3.50/4.05
	3.50	70.1	
	4.05	58.3	
**Cytosine**	7.51	146.3	5.96/7.49
	-	-	7.50/5.97
**Ethanol**	1.17	19.6	-
	3.64	60.3	
**Formate**	8.45	173.8	8.45/8.45
**Fucose***	5.11	97.7	3.92/3.65
	3.92	73.5	3.44/3.88
	3.71	73.0	
	3.43	74.6	
	3.87	70.8	
	4.88	97.7	
	3.63	75.6	
	-	-	
	-	-	
**Fumarate**	6.51	138.1	-
**Galactose**	5.26	95.1	
	4.57	99.3	
	4.07	73.3	
	3.98	72.1	
	3.92	71.5	
	3.85	72.1	-
	3.80	71.0	
	3.75	63.8	
	3.70	77.9	
	3.63	75.6	
	3.48	74.8	
**Glucose**	5.22	94.9	
	-	-	
	3.90	63.4	
	3.82	63.3	
	3.81	74.1	
	3.72	63.5	-
	3.70	75.6	
	3.52	74.3	
	3.45	78.6	
	3.40	72.5	
	3.23	77.0	
**Glutamate**	2.11	29.8	3.75/2.05
	2.34	36.2	3.75/2.34
	3.75	57.5	2.12/2.34
**Glutamine**	2.11	29.8	3.75/2.34
	2.34	36.2	2.12/2.34
	3.75	57.5	2.06/3.75
**Glycerol**	3.55	65.4	
	3.65	65.4	-
	3.80	74.8	
**Glycine**	3.55	44.4	-
**Histidine**	3.18	30.3	7.13/8.00
	3.27	30.2	7.13/3.26
	3.28	29.3	7.13/3.18
	4.00	57.3	4.00/3.26
	7.14	120.0	4.00/3.18
	8.03	138.4	
**Isoleucine**	0.93	13.8	
	1.0	17.4	
	1.45	27.0	-
	1.96	38.7	
	3.65	62.5	
**Lactate**	1.31	22.9	-
	4.15	71.1	
**Leucine**	0.94	23.7	
	0.95	24.7	
	1.69	26.9	-
	1.72	42.6	
	3.73	56.4	
**Lysine**	1.43	24.1	
	1.49	24.2	
	1.7	29.1	-
	1.89	32.7	
	3.04	41.8	
	3.75	57.5	
**Methanol**	3.35	51.8	-
**Methionine**	2.12	16.6	
	2.18	32.6	-
	2.63	31.5	
	3.85	56.8	
**Ornithine**	3.75	57.2	3.04/1.76
	3.04	41.8	3.04/1.93
	1.93	30.2	3.04/3.75
	-	-	1.93/1.77
**Phenylalanine**	3.12	39.2	
	3.28	39.2	
	3.99	58.9	-
	7.32	132.1	
	7.37	130.5	
	7.42	131.9	
**Proline**	1.99	26.5	
	2.05	31.7	-
	2.33	31.7	
	3.33	49.0	
	3.41	49.0	
	4.12	64.0	
**Propionate**	2.17	33.4	-
	1.04	12.9	
**Putrescine**	3.04	41.8	1.76/3.04
	1.76	26.7	
**Pyruvate**	2.36	29.1	-
**Serine**	3.97	63.0	-
	3.83	59.3	
**Succinate**	2.39	36.8	-
**Taurine**	3.25	50.4	3.41/3.25
	3.42	38.3	
**Tryptophan**	3.28	29.2	
	3.49	29.1	
	4.05	58.3	
	7.19	122.0	-
	7.27	124.9	
	7.31	128.0	
	7.53	114.8	
	7.72	121.3	
**Tyrosine**	3.04	38.3	3.17/3.95 7.17/6.89
	3.19	38.3	
	3.93	59.0	
	6.89	118.7	
	7.20	133.1	
**Uracil**	7.52	146.4	5.79/7.53
	-	-	7.52/5.79
	5.79	103.8	
**Valine**	0.98	19.4	
	1.03	20.7	-
	2.26	31.9	
	3.60	63.3	
**Xanthine**	7.74	140.1	7.72/7.72
***myo*-Inositol**	3.27	77.2	4.05/3.51
	3.53	73.9	4.05/3.26
	3.63	75.9	3.27/3.52
	4.05	75.1	3.63/3.28
			3.60/4.05

## Appendix B

Concentration tables and ANOVA tables of metabolites detected by NMR in infant fecal concentrations. An asterisk (*) signifies significance in a comparison between fermentation of sheep milk and cow milk, an obelisk (†) signifies significance in a comparison between fermentation of sheep milk and the control (buffer), and a dagger (‡) signifies significance in a comparison between fermentation of cow milk and the control (buffer).

**Table A3:** Concentrations of metabolites detected by NMR in one-month-old fecal fermentations.

	CM (mM)			SM (mM)			Control (mM)		
Metabolite	4 h	7 h	24 h	4 h	7 h	24 h	4 h	7 h	24 h
Acetate †‡	333.03	431.38	2263.06	376.31	509.63	1998.08	137.56	194.41	1024.92
Alanine * †‡	52.84	55.22	74.85	39.02	43.77	59.19	10.18	11.36	17.10
Arginine *†‡	32.74	31.30	1.65	24.57	24.94	3.11	0.00	0.00	0.00
Aspartate †‡	33.13	35.03	26.04	26.46	37.30	22.59	10.71	9.93	6.73
Butyrate †‡	9.32	16.06	92.74	5.07	30.26	92.45	3.01	8.37	54.70
Choline *†‡	5.50	5.56	7.06	4.55	4.62	4.98	1.67	1.62	1.20
Ethanol *†	305.87	165.23	122.26	296.97	353.30	205.72	217.06	240.41	103.20
Formate *†‡	168.20	197.60	298.75	163.34	227.03	350.43	9.38	23.70	103.39
Fucose *†‡	27.81	29.05	148.65	25.60	28.89	99.15	28.48	23.08	60.59
Galactose *	18.98	11.71	2.95	2.89	2.71	0.93	2.46	39.13	1.04
Glucose-6-phosphate *†‡	111.33	52.24	28.57	100.23	102.24	57.16	68.81	38.29	20.52
Glutamate †‡	48.66	55.38	18.74	41.23	50.69	16.02	15.70	17.02	0.00
Glycine †‡	74.39	84.66	73.48	72.79	76.23	64.95	24.02	12.47	11.44
Isoleucine *†‡	64.17	52.66	30.61	44.10	42.26	18.61	7.78	6.65	1.14
Lactate †‡	98.88	119.49	19.58	103.46	126.70	17.68	34.32	36.55	35.69
Lactose *†	46.53	119.05	38.56	37.10	35.64	23.74	33.91	36.94	105.91
Leucine *†‡	62.01	63.57	24.80	39.85	39.85	7.81	7.50	8.65	3.07
Lysine *†‡	53.98	50.12	50.32	36.53	27.39	21.21	0.00	0.00	3.41
Methanol †‡	48.86	48.29	49.55	47.79	48.24	49.36	50.71	50.27	49.94
Methionine †‡	16.07	10.59	0.90	15.20	6.95	4.99	0.00	0.00	1.95
myo-Inositol	8.31	7.97	0.00	0.00	0.00	0.00	0.00	0.00	5.76
N-Acetylglucosamine *‡	35.94	11.68	15.28	11.38	2.76	9.63	7.34	10.34	9.68
N-acetylmannosamine *‡	34.27	38.17	6.13	37.79	40.11	18.67	34.55	40.60	31.10
Ornithine ‡	0.00	0.00	15.62	1.57	2.85	7.84	0.00	0.00	2.15
Phenylalanine *†‡	19.34	20.46	13.38	13.32	12.86	9.45	0.00	0.00	0.37
Proline *‡	19.68	17.53	46.09	6.87	9.38	5.98	0.00	0.00	2.08
Propionate *†‡	1.29	9.91	346.98	2.95	10.08	254.29	2.47	6.13	105.14
Pyruvate †‡	30.99	25.84	0.00	34.56	16.35	2.21	18.41	16.05	0.00
Succinate *†‡	12.71	23.91	1377.31	13.04	32.25	1053.40	9.29	16.82	386.52
Threonine †‡	41.95	25.67	5.15	26.65	15.75	9.47	1.15	7.39	4.46
Tryptophan *†‡	13.08	13.06	10.80	9.15	9.43	7.80	2.20	1.70	0.82
Tyrosine *†‡	25.36	25.55	0.00	14.27	16.74	2.39	4.12	4.63	0.97
Uracil †‡	3.89	8.26	10.04	2.01	9.24	10.36	1.71	1.95	0.00
Valine *†‡	62.09	65.16	57.34	44.28	48.48	35.19	9.51	9.63	8.74

**Table A4:** Metabolites detected by NMR in one-month-old fecal fermentations, analyzed using ANOVA.

	CM vs. SM				CM vs. Control				SM vs. Control			
	Substrate		Time		Substrate		Time		Substrate		Time	
Metabolite	*p* Value	fdr	*p* Value	fdr	*p* Value	fdr	*p* Value	fdr	*p* Value	fdr	*p* Value	fdr
Acetate †‡	0.351	0.459	0.000	0.001	0.000	0.000	0.000	0.000	0.000	0.000	0.000	0.000
Alanine *†‡	0.000	0.000	0.000	0.000	0.000	0.000	0.000	0.000	0.000	0.000	0.000	0.000
Arginine *†‡	0.035	0.057	0.000	0.000	0.000	0.001	0.001	0.003	0.000	0.000	0.001	0.002
Aspartate †‡	0.192	0.283	0.000	0.001	0.000	0.000	0.014	0.019	0.000	0.000	0.002	0.004
Butyrate †‡	0.521	0.611	0.000	0.000	0.000	0.000	0.000	0.000	0.000	0.000	0.000	0.000
Choline *†‡	0.000	0.000	0.004	0.008	0.000	0.000	0.003	0.005	0.000	0.000	1.000	1.000
Ethanol *†	0.019	0.037	0.018	0.022	0.804	0.828	0.009	0.014	0.002	0.002	0.001	0.002
Formate *†‡	0.006	0.014	0.000	0.000	0.000	0.000	0.000	0.000	0.000	0.000	0.000	0.000
Fucose *†‡	0.000	0.001	0.000	0.000	0.000	0.001	0.000	0.000	0.001	0.001	0.000	0.000
Galactose *	0.000	0.001	0.009	0.013	1.000	1.000	0.295	0.334	0.482	0.512	0.278	0.364
Glucose-6-phosphate *†‡	0.025	0.044	0.000	0.001	0.048	0.054	0.000	0.000	0.001	0.001	0.008	0.015
Glutamate †‡	0.594	0.652	0.015	0.019	0.005	0.006	0.019	0.026	0.001	0.001	0.010	0.017
Glycine †‡	0.226	0.320	0.184	0.195	0.000	0.000	0.845	0.898	0.000	0.000	0.692	0.800
Isoleucine *†‡	0.000	0.000	0.000	0.000	0.000	0.000	0.000	0.000	0.000	0.000	0.000	0.001
Lactate †‡	0.556	0.630	0.000	0.000	0.000	0.000	0.000	0.000	0.000	0.000	0.000	0.000
Lactose *†	0.000	0.001	0.003	0.005	0.228	0.250	0.005	0.008	0.002	0.002	0.014	0.024
Leucine *†‡	0.000	0.000	0.000	0.000	0.000	0.000	0.000	0.000	0.000	0.000	0.000	0.000
Lysine *†‡	0.001	0.003	0.737	0.759	0.000	0.000	0.980	1.000	0.000	0.001	0.665	0.800
Methanol †‡	0.380	0.478	0.167	0.183	0.033	0.039	0.706	0.774	0.007	0.009	0.760	0.834
Methionine †‡	1.000	1.000	0.009	0.013	0.002	0.003	0.072	0.088	0.001	0.001	0.084	0.119
myo-Inositol	0.304	0.414	1.000	1.000	0.725	0.771	1.000	1.000	1.000	1.000	1.000	1.000
N-Acetylglucosamine *‡	0.013	0.025	0.050	0.058	0.012	0.015	0.108	0.127	0.633	0.653	0.706	0.800
N-acetylmannosamine *‡	0.000	0.000	0.000	0.000	0.008	0.010	0.001	0.002	0.182	0.200	0.001	0.003
Ornithine ‡	0.902	0.929	0.007	0.011	0.016	0.019	0.000	0.001	0.035	0.041	0.075	0.111
Phenylalanine *†‡	0.000	0.000	0.000	0.000	0.000	0.000	0.000	0.000	0.000	0.000	0.195	0.265
Proline *‡	0.001	0.002	0.053	0.060	0.000	0.000	0.006	0.009	0.041	0.047	1.000	1.000
Propionate *†‡	0.007	0.015	0.000	0.000	0.000	0.000	0.000	0.000	0.000	0.000	0.000	0.000
Pyruvate †‡	0.505	0.611	0.000	0.000	0.000	0.000	0.000	0.000	0.004	0.005	0.000	0.000
Succinate *†‡	0.026	0.044	0.000	0.000	0.000	0.000	0.000	0.000	0.000	0.000	0.000	0.000
Threonine †‡	0.173	0.267	0.013	0.018	0.004	0.005	0.056	0.070	0.008	0.010	0.374	0.471
Tryptophan *†‡	0.000	0.000	0.006	0.009	0.000	0.000	0.001	0.002	0.000	0.000	0.024	0.039
Tyrosine *†‡	0.004	0.010	0.000	0.000	0.000	0.000	0.000	0.000	0.000	0.000	0.000	0.000
Uracil †‡	0.804	0.854	0.005	0.008	0.000	0.000	0.029	0.038	0.000	0.000	0.001	0.002
Valine *†‡	0.000	0.000	0.015	0.019	0.000	0.000	0.001	0.001	0.000	0.000	0.067	0.103

**Table A5:** Concentrations of metabolites detected by NMR in five-month-old fecal fermentations.

	CM (mM)			SM (mM)			Control (mM)		
Metabolite	4 h	7 h	24 h	4 h	7 h	24 h	4 h	7 h	24 h
5,6-Dihydrouracil	0.00	0.00	14.32	0.00	0.00	14.87	0.00	0.00	5.92
Acetate *†‡	507.95	800.65	1154.94	563.61	776.46	1272.14	283.88	412.59	696.04
Alanine †‡	61.21	72.60	25.66	58.40	69.24	31.14	19.44	21.39	23.29
Arginine	0.00	0.00	0.00	0.00	0.00	5.42	0.00	0.00	0.00
Aspartate †‡	57.36	6.93	0.00	51.30	5.61	0.00	12.26	2.14	0.00
Betaine	1.86	1.43	1.63	1.48	2.30	1.79	1.19	1.52	1.63
Choline †‡	12.41	14.02	15.06	11.90	13.20	15.25	9.09	8.96	9.66
Cytosine	0.00	0.00	0.00	0.00	0.00	0.00	0.32	0.00	0.00
Ethanol †‡	44.75	93.97	115.48	70.25	93.62	183.88	37.23	48.60	56.75
Formate †‡	76.42	251.18	126.74	137.67	235.83	150.00	33.27	88.16	56.28
Fucose	13.32	0.00	0.00	0.00	0.00	0.00	19.96	0.00	0.00
Fumarate	1.39	2.40	0.00	2.24	1.89	0.00	2.00	1.08	0.07
Galactose †	8.59	10.84	4.90	8.45	11.16	14.80	5.91	7.33	2.08
Glucose ‡	14.16	4.22	0.54	2.38	4.82	8.75	2.25	0.84	0.00
Glutamate †‡	65.03	69.47	79.47	55.88	60.37	74.79	13.08	14.54	10.01
Glutamine *†‡	27.49	23.84	0.00	18.98	18.60	0.00	0.00	0.00	0.00
Glycerol †	19.10	13.21	0.00	0.00	12.61	0.00	30.34	14.62	0.00
Glycine †‡	70.13	85.82	92.04	69.10	85.07	93.55	18.52	20.71	4.32
Histidine *†‡	11.08	17.63	18.73	9.23	12.15	16.85	1.58	1.88	1.10
Hydroxyacetone	8.45	2.18	1.60	5.94	5.04	5.14	2.49	2.89	2.14
Isoleucine *†‡	52.86	57.70	56.78	41.57	50.77	47.15	8.46	8.39	6.29
Lactate †‡	221.90	228.48	110.60	232.12	225.88	90.17	82.95	82.68	27.59
Leucine *†‡	58.56	66.12	62.90	45.08	55.02	46.92	8.67	11.37	9.04
Lysine †‡	37.87	27.87	10.49	51.67	31.99	14.69	0.00	0.00	0.00
Methanol	6.12	3.72	5.67	3.95	4.35	5.62	4.42	3.85	5.47
Methionine *†‡	14.23	14.90	16.52	10.31	12.99	12.40	2.07	3.25	2.35
myo-Inositol	0.00	3.85	15.40	14.41	0.00	0.00	7.21	6.68	8.01
Ornithine †‡	10.05	4.50	0.00	11.51	6.38	0.00	0.95	0.52	0.53
Phenylalanine †‡	5.08	18.90	21.31	4.66	14.99	21.16	0.00	0.85	0.00
Proline †‡	21.06	36.38	64.35	16.35	26.12	72.17	0.00	1.84	6.11
Propionate †‡	9.57	41.34	372.47	10.92	33.22	392.88	9.23	15.63	115.11
Putrescine †‡	0.00	0.00	34.33	0.00	0.00	35.58	0.00	0.00	0.00
Pyruvate †‡	33.90	0.61	0.00	35.85	0.12	0.00	9.37	0.20	0.00
Serine	28.16	0.00	0.00	0.00	0.00	0.00	0.00	0.00	0.00
Succinate †‡	44.09	138.78	214.41	48.71	129.11	215.28	43.09	71.74	122.43
Taurine	0.00	0.58	0.00	0.00	0.00	0.00	2.82	5.26	0.00
Tryptophan †‡	10.04	13.06	4.12	10.62	15.11	5.13	1.84	2.15	0.91
Tyrosine †‡	23.12	28.32	7.79	14.79	25.78	2.75	4.00	4.22	1.24
Uracil †‡	12.98	14.91	4.35	13.99	15.93	3.71	6.13	6.46	1.59
Valine *†‡	67.25	75.64	85.47	53.75	68.35	74.10	10.03	7.87	10.06
Xanthine *‡	1.34	13.17	0.00	0.00	4.44	0.00	2.83	3.67	0.00

**Table A6:** Metabolites detected by NMR in five-month-old fecal fermentations, analyzed using ANOVA.

	CM vs. SM				CM vs. Control				SM vs. Control			
	Substrate		Time		Substrate		Time		Substrate		Time	
Metabolite	*p* Value	fdr	*p* Value	fdr	*p* Value	fdr	*p* Value	fdr	*p* Value	fdr	*p* Value	fdr
5,6-Dihydrouracil	0.573	0.937	0.017	0.031	0.241	0.308	0.018	0.036	0.330	0.387	0.027	0.055
Acetate *†‡	0.013	0.122	0.000	0.000	0.000	0.000	0.000	0.000	0.000	0.000	0.000	0.000
Alanine †‡	1.000	1.000	0.006	0.012	0.000	0.000	0.023	0.042	0.002	0.004	0.108	0.159
Arginine	1.000	1.000	1.000	1.000	1.000	1.000	1.000	1.000	1.000	1.000	1.000	1.000
Aspartate †‡	0.413	0.839	0.000	0.000	0.000	0.000	0.000	0.000	0.000	0.000	0.000	0.000
Betaine	0.215	0.679	0.516	0.622	0.385	0.464	0.824	0.891	0.081	0.110	0.113	0.159
Choline †‡	0.311	0.839	0.000	0.001	0.000	0.000	0.006	0.015	0.000	0.000	0.001	0.003
Cytosine	1.000	1.000	1.000	1.000	1.000	1.000	1.000	1.000	1.000	1.000	1.000	1.000
Ethanol †‡	0.161	0.611	0.002	0.004	0.000	0.000	0.001	0.002	0.000	0.000	0.016	0.037
Formate †‡	0.148	0.611	0.000	0.000	0.000	0.001	0.003	0.009	0.000	0.000	0.007	0.019
Fucose	1.000	1.000	1.000	1.000	0.824	0.913	0.083	0.117	0.186	0.231	0.165	0.218
Fumarate	0.594	0.937	0.000	0.001	0.324	0.402	0.000	0.000	0.364	0.414	0.001	0.002
Galactose †	0.366	0.839	0.944	1.000	0.183	0.242	0.102	0.140	0.025	0.038	0.948	1.000
Glucose ‡	0.784	1.000	0.697	0.816	0.003	0.005	0.005	0.013	0.126	0.161	0.918	1.000
Glutamate †‡	0.179	0.611	0.046	0.073	0.000	0.000	0.557	0.634	0.000	0.000	0.073	0.110
Glutamine *†‡	0.012	0.122	0.000	0.000	0.000	0.000	0.000	0.001	0.000	0.000	0.000	0.000
Glycerol †	0.405	0.839	0.327	0.432	0.452	0.529	0.042	0.063	0.040	0.059	0.053	0.087
Glycine †‡	1.000	1.000	0.001	0.002	0.000	0.000	0.024	0.043	0.000	0.000	0.023	0.049
Histidine *†‡	0.031	0.179	0.002	0.004	0.000	0.000	0.069	0.101	0.000	0.000	0.007	0.019
Hydroxyacetone	0.373	0.839	0.075	0.112	0.053	0.075	0.006	0.014	0.063	0.089	0.922	1.000
Isoleucine *†‡	0.018	0.122	0.227	0.310	0.000	0.000	0.826	0.891	0.000	0.000	0.221	0.275
Lactate †‡	0.745	0.985	0.000	0.000	0.000	0.000	0.000	0.000	0.000	0.000	0.000	0.000
Leucine *†‡	0.004	0.074	0.363	0.451	0.000	0.000	0.163	0.216	0.000	0.000	0.311	0.364
Lysine †‡	0.581	0.937	0.076	0.112	0.010	0.015	0.463	0.542	0.001	0.001	0.071	0.110
Methanol	0.667	0.970	0.347	0.445	0.765	0.871	0.269	0.324	0.725	0.804	0.038	0.071
Methionine *†‡	0.001	0.033	0.103	0.145	0.000	0.000	0.211	0.262	0.000	0.000	0.180	0.231
myo-Inositol	0.300	0.839	0.033	0.054	0.902	0.973	0.021	0.041	0.192	0.231	0.014	0.033
Ornithine †‡	0.745	0.985	0.013	0.024	0.021	0.031	0.036	0.057	0.019	0.029	0.041	0.073
Phenylalanine †‡	0.686	0.970	0.025	0.043	0.000	0.000	0.013	0.027	0.007	0.012	0.142	0.194
Proline †‡	0.505	0.937	0.000	0.000	0.000	0.000	0.000	0.000	0.000	0.000	0.000	0.000
Propionate †‡	0.843	1.000	0.000	0.001	0.000	0.000	0.000	0.000	0.002	0.003	0.000	0.000
Putrescine †‡	0.556	0.937	0.000	0.000	0.004	0.006	0.004	0.010	0.011	0.019	0.011	0.029
Pyruvate †‡	0.623	0.946	0.000	0.001	0.000	0.000	0.000	0.000	0.000	0.000	0.000	0.000
Serine	1.000	1.000	1.000	1.000	1.000	1.000	1.000	1.000	1.000	1.000	1.000	1.000
Succinate †‡	1.000	1.000	0.000	0.000	0.000	0.000	0.000	0.000	0.000	0.000	0.000	0.000
Taurine	1.000	1.000	1.000	1.000	0.128	0.174	0.178	0.229	0.122	0.161	0.254	0.306
Tryptophan †‡	0.430	0.839	0.002	0.004	0.001	0.001	0.027	0.046	0.000	0.000	0.006	0.019
Tyrosine †‡	0.171	0.611	0.000	0.000	0.000	0.000	0.001	0.003	0.000	0.000	0.004	0.013
Uracil †‡	0.382	0.839	0.000	0.000	0.000	0.000	0.000	0.000	0.000	0.000	0.000	0.000
Valine *†‡	0.015	0.122	0.004	0.008	0.000	0.000	0.036	0.057	0.000	0.000	0.045	0.077
Xanthine *‡	0.035	0.179	0.002	0.005	0.002	0.004	0.000	0.000	0.863	0.931	0.033	0.065
